# Production, Purification, and Potential Health Applications of Edible Seeds’ Bioactive Peptides: A Concise Review

**DOI:** 10.3390/foods10112696

**Published:** 2021-11-04

**Authors:** Mrinal Samtiya, Sovon Acharya, Kush Kumar Pandey, Rotimi E. Aluko, Chibuike C. Udenigwe, Tejpal Dhewa

**Affiliations:** 1 Department of Nutrition Biology, School of Interdisciplinary and Applied Sciences, Central University of Haryana, Mahendergarh 123031, India; mrinalsamtiya@gmail.com; 2 Research and Development Unit, Abiocis Bio-Science Pvt. Ltd., Hyderabad 500026, India; sovonacharya@gmail.com (S.A.); kushpandey0308@gmail.com (K.K.P.); 3 Department of Food and Human Nutritional Sciences, University of Manitoba, Winnipeg, MB R3T 2N2, Canada; 4 School of Nutrition Sciences, Faculty of Health Sciences, University of Ottawa, Ottawa, ON K1H 8M5, Canada; cudenigw@uottawa.ca

**Keywords:** bioactive peptides, protein hydrolysates, health attributes, edible seeds, antihypertensive, anti-cancer

## Abstract

Edible seeds play a significant role in contributing essential nutritional needs and impart several health benefits to improve the quality of human life. Previous literature evidence has confirmed that edible seed proteins, their enzymatic hydrolysates, and bioactive peptides (BAPs) have proven and potential attributes to ameliorate numerous chronic disorders through the modulation of activities of several molecular markers. Edible seed-derived proteins and peptides have gained much interest from researchers worldwide as ingredients to formulate therapeutic functional foods and nutraceuticals. In this review, four main methods are discussed (enzymatic hydrolysis, gastrointestinal digestion, fermentation, and genetic engineering) that are used for the production of BAPs, including their purification and characterization. This article’s main aim is to provide current knowledge regarding several health-promoting properties of edible seed BAPs in terms of antihypertensive, anti-cancer, antioxidative, anti-inflammatory, and hypoglycemic activities.

## 1. Introduction

Edible seeds such as cereals and pulses form a significant part of the human diet. As suggested by the Food and Agricultural Organization (FAO), only a few quantities (approx. a few hundred) of plant species are consumed as main food sources, out of approx. 50,000 edible plant species [1]. Moreover, edible seeds contain a diverse range of phytochemicals with numerous important biological activities [2]. Currently, consumers want healthy, functional foods (such as vegetables, fruits, seeds, and nuts) as part of their dietary necessity to sustain a healthy human lifestyle. This is due to the increased incidence of several non-communicable disorders (NCDs) such as cancer, cognitive impairment, metabolic syndrome, high blood pressure, diabetes, and cardiovascular disease (CVD), which could be prevented or ameliorated through changes in dietary lifestyles [3]. NCDs such as diabetes, chronic respiratory disease, cancer, and CVDs have contributed to approximately 41 million deaths, i.e., 71% of total global deaths [4]. Usually, NCDs are associated with various root causes and are characterized by slow but progressive body degeneration, which significantly reduces the quality of life (development of infirmities) and eventually can be fatal [5]. Bioactive peptides (BAPs) are proteins that positively influence human health via certain cellular metabolic processes and hence are designated as bioactive components [6]. BAPs are generally comprised of 2 to 20 amino acid (AA) residues, which could be produced through microbial fermentation and protein hydrolysis by endogenous or exogenous proteases [7]. Protein precursors of BAPs can be classified according to plant, marine, and animal origins. Several works have examined the therapeutic attributes of BAPs that are derived from marine sources (seahorse, oyster, salmon, and fish) and other animal sources such as meat, muscle, egg, and milk [8]. However, recent processing technologies and human nutrition have focused on plant proteins as a significant source of food-derived bioactive compounds [9]. Among these bioactive compounds, food-derived BAPs are potentially active components that can be used to develop nutraceuticals and functional foods based on their presumed numerous health benefits and safety [10,11]. BAPs are inactive when present as an integral part of the parent protein and need to be released through peptide bond hydrolysis to impart health benefits [12,13]. BAPs are recognized as the components that can interact with and regulate the biological functions of specific cellular receptors and biomolecules [9,14]. Several potential properties of BAPs have been reported, with confirmation in animal, cell culture, and in vitro studies, including anti-adhesive, anti-cancer, anti-diabetic, lipid-lowering, immunomodulatory, anti-inflammatory, antioxidative, and antihypertensive properties [7,15,16]. Moreover, these attributes are mainly dependent on the peptide size and molecular weight in addition to AA sequence and type [17,18,19]. Additionally, the antioxidative characteristics of peptides are based on their hydrophobic nature, structural conformation, and AA composition. For example, valine, threonine, phenylalanine, isoleucine, leucine, glycine, lysine, cysteine, methionine, histidine, tyrosine, glutamic acid, and proline contribute positively to the antioxidant activity of peptides [20,21,22]. Structural characteristics of antimicrobial BAPs can be considered as amphipathic, basic (arginine or lysine-rich), and small (≤20 to 46 AAs) peptides [23,24]. With respect to the mode of action, the anti-carcinogenic properties of BAPs could be attributed to the inhibition of cancer cells’ intracellular signaling, immune response modulation, topoisomerases inhibition, cell adhesion, and cell membrane structure disruption [25]. Peptides that inhibit angiotensin-converting enzyme (ACE) activity have vital physiological functions in maintaining normal body fluid, salt balance, and blood pressure [19]. The immunomodulatory property of BAPs is mainly dependent on cytokine regulation and control in addition to the stimulation of antibody production and immune system development [8]. Therefore, various food-derived BAPs and proteins could enhance human health by preventing several ailments and mitigating chronic diseases [26]. BAPs can be produced using enzymatic hydrolysis of food proteins to liberate peptide sequences, which can be subsequently subjected to post-hydrolysis processing to separate inactive from active peptides [8]. These peptides are embedded as AA (inactive) sequences in the primary structure of animal and plant proteins and could be liberated during gastrointestinal digestion, in vitro enzyme-catalyzed proteolysis, and fermentation [8,27]. Although the mass production of BAPs is feasible through in vitro enzymatic hydrolysis of proteins, recombinant DNA technology is also being explored [28,29], particularly to produce proteins and long-chain peptides [30,31]. In the following sections, specific health-promoting potentials of peptides that are derived from edible plant seeds, as well as their possible mechanisms of action, are discussed. In addition, information is provided on the production, identification, and characterization methods used to discover these BAPs. The main aim of this review is to provide sufficient knowledge of beneficial health attributes of BAPs (i.e., antioxidant and anti-inflammatory activities, antihypertensive activity, hypoglycemic activity, anti-cancer activity, and mineral-binding peptides) that are derived from edible seeds. This information could be useful for researchers who are working in the nutraceutical and functional foods research area.

## 2. Research Methods

For this article, research and review papers were collected using Scopus electronic databases, Google Scholar, and PubMed until June 2021. The particular keywords used for searches were: “edible seeds bioactive peptides”; “bioactive peptides health benefits”; “production methods of bioactive peptides”; “antihypertensive activity”; “anti-cancer activity”; among others. All the references that are included were manually selected and reviewed for appropriate content.

## 3. Production of Bioactive Peptides

BAPs can be produced by several processes, such as gastrointestinal digestion, fermentation, and enzymatic hydrolysis [25]. Furthermore, it is also possible to produce peptides through chemical synthesis or through genetic recombination using microorganisms if the peptide sequence is already identified [32]. In addition, BAPs can be purified, isolated for AA sequencing and synthesis, and subsequently used to prepare nutraceuticals [14]. This section provides an overview of the major processes used for the sustainable production of BAPs from edible seed proteins.

### 3.1. Enzymatic Hydrolysis

BAPs can be produced from edible seeds through enzymatic hydrolysis of the protein using proteinases such as thermolysin, savinase, flavourzyme, elastase, trypsin, pepsin, chymotrypsin, alcalase, and pancreatin alone or in various combinations [33,34,35,36,37]. Hydrolysates of food proteins are frequently achieved via digestion (enzymatic) followed by subsequent separation of the peptides (soluble phase) from undigested proteins (insoluble phase) [38]. Protein hydrolysis is commonly regulated by the release of H^+^ (proton) that builds up and reduces the pH of the mixture as the enzymatic reaction progresses with time. However, excessive pH reduction must be avoided because it can negatively affect the optimal rate of hydrolysis by causing the inactivation of proteases. Therefore, to maintain the optimum pH level, a suitable base such as NaOH can be added during protein hydrolysis to neutralize the released protons [39]. Upon the completion of enzymatic hydrolysis, the reaction mixture is centrifuged to obtain a supernatant, which can be subjected to peptide fractionation or dried to obtain the protein hydrolysate. For example, sesame peptides were prepared by hydrolyzing sesame seed protein using papain. The degree of hydrolysis was 14.98% when the optimum conditions were pH 9.0, 1500 U/g enzyme activity, 4% (*w*/*v*) substrate concentration, 60 °C temperature, and a reaction time of 3 h [40]. A study by Olagunju et al. [41] assessed the potential antioxidant properties of pigeon pea protein after hydrolysis with alcalase, pancreatin, and pepsin + pancreatin. The results showed that the fraction with <1 kDa molecular weight had the highest peptide yield of 36.97% for pepsin + pancreatin hydrolysates. Another study on flaxseed peptides obtained at two concentrations (2.5 and 3.0%, *w*/*w*) of thermoase protease reported that the highest antioxidative attributes were for the fractions obtained with 2.5% [42]. These studies demonstrate a need to optimize downstream processing, including the type of enzyme, reaction conditions, and post-hydrolysis processing methods, to obtain the highest yield of protein hydrolysates and peptides with strong bioactivities.

### 3.2. Gastrointestinal Digestion

During hydrolysis, several enzymes that are derived from various sources, such as microorganisms, can be used to produce BAPs. However, for simulated gastrointestinal tract (GIT) digestion, only enzymes native to the human digestive tract are used under similar conditions (temperature, pH, etc.) that prevail in the GIT. During GIT hydrolysis, BAPs can be liberated from food proteins via the action of digestive enzymes such as amino- and carboxypeptidases, chymotrypsin, trypsin, and pepsin [33,43]. The acidic conditions in the stomach can denature food proteins and enhance subsequent proteolysis. To simulate GIT digestion, the most used enzymes are pepsin and pancreatin for sequential protein hydrolysis. In general, the protein solution is initially adjusted to pH 2.0 and 37 °C, and subsequently, pepsin is added to digest the sample for 1–3 h, which simulates the stomach phase of digestion. After pepsin digestion, the whole reaction mixture is adjusted to pH 7.0–7.5, and pancreatin digestion is conducted for 3–4 h to simulate the intestinal phase [44,45]. Capriotti et al. [46] reported that many peptides were formed after in vitro gastrointestinal digestion of soybean proteins.

Moreover, Wang et al. [47] isolated peptides from sesame seed protein by means of in vitro GIT digestion with α-chymotrypsin, trypsin, and pepsin. Furthermore, 23 peptides were generated from hemp seed proteins by in vitro GIT digestion [48]. Despite the prospects of this method, it is worth noting that the peptide profiles and bioactivity may not necessarily be replicated during in vivo digestion by humans due to the complex nature of the GIT when compared to isolated in vitro digestion models.

### 3.3. Fermentation

Fermentation is a method that uses the proteolytic systems of microorganisms for BAP production [10]. Microorganisms used in fermentation can secrete a range of proteases, which hydrolyze proteins into simple peptides [49]. For example, during fermentation, bacteria/microbes hydrolyze the proteins into peptides and AAs to produce nitrogen sources required for their development [50]. The process can be controlled to avoid complete digestion. The resulting peptides can be collected by centrifugation [51], separated via molecular sieve or ultrafiltration, and purified through chromatographic techniques for sequencing and bioactivity evaluation [52,53]. A recent study by Ayyash et al. [54] reported on the fermentation of quinoa seed flour by *Lactobacillus* spp. For 72 h, protein hydrolysis increased by 11.5–30.0%, which enhanced the contents of AAs and small peptides. Likewise, the solid-state fermentation of five common pulses (chickpea, faba bean, kidney bean, green lentil, and yellow pea) with *L. plantarum* resulted in a significant increase in their simulated GIT digestion, as reflected by the high degree of hydrolysis [55]. These effects were more pronounced in flours than intact seeds due to the increased surface area and contact between the microbial proteases and legume flour matrix proteins. Previous evidence by Rizzello et al. [56] confirmed that during lactic acid fermentation of amaranth flour, lunasin, a cancer-preventive peptide with 43 AA residues, was generated. In the study, amaranth flour was inoculated with each of the five strains of lactic acid bacteria (*L. plantarum* 3DM, *L. pentosus* 12H6, *L. brevis* AM7, *L. rossiae* CD76, and *L. curvatus* SAL33) that contain peptidase activities for 16 h at 30 °C at a cell density of 8.0 log colony forming units (CFU)/g. Furthermore, a study reported that the solid-state fermentation of red beans with *Cordyceps militaris* for seven days at 25 °C increased proteins, small peptides, essential AAs, and in vitro protein digestibility by 6.54% when compared to the non-fermented beans [57]. These effects are thought to be due to the activities of proteases formed by microorganisms. However, there is limited information on the specificities of microbial proteases released during fermentation, making it challenging to predict and design the cleavage patterns and release of BAPs from edible seed proteins.

### 3.4. Genetic Engineering

Recombinant DNA technology is being explored for the large-scale production of BAPs, particularly for long-chain peptides [32]. Over the years, microbial production has made it possible to adapt small peptides after natural isolation to laboratory research by engineering the biosynthetic pathway using cutting-edge emerging approaches and synthetic biology. This process includes the synthesis of peptides’ coding region and consequent cloning through prokaryotic or eukaryotic expression vectors using *E. coli* or yeast as host cells. This cloning approach permits the construction and expression of more than one peptide concurrently [58]. For example, a recent work reported the successful simultaneous cloning and expression of ten ACE-inhibitory peptides (DKIHPF, YQQPVL, IPP, VPP, LKPNM, RPLKPW, KVLPVPE, SKVYPFPGPI, YLAHKALPMHIR, and FFVAPFPEVFGK) in rice grain [59]. Likewise, three soy-derived peptides, YPLDLF, LPYPR, and RPLKPW, which possessed memory-enhancing, hypocholesterolemic, and hypotensive activities, respectively, were expressed in transgenic plants [60]. Furthermore, transgenic rice was used to produce RPLKPW with the rice storage protein glutelin as a fusion protein, and, under the promoter regulation of endosperm-specific glutelin, the engineered peptide was expressed in the seeds. The authors reported that the transgenic rice seeds and RPLKPW-glutelin fraction significantly reduced the systolic blood pressure (SBP) of spontaneously hypertensive rats after oral administration [61]. This indicates that the proteolytic machinery of the GIT may be capable of in vivo digestion and release of the bioactive peptide(s) cloned into the protein sequence. Although this method of producing BAPs appears promising, the ongoing global debate and consumer concern about genetically modified foods may delay its application in the functional foods industry compared to bioprocessing (enzymatic and fermentation) methods.

## 4. Purification and Characterization of Bioactive Peptides

**Figure 1:**
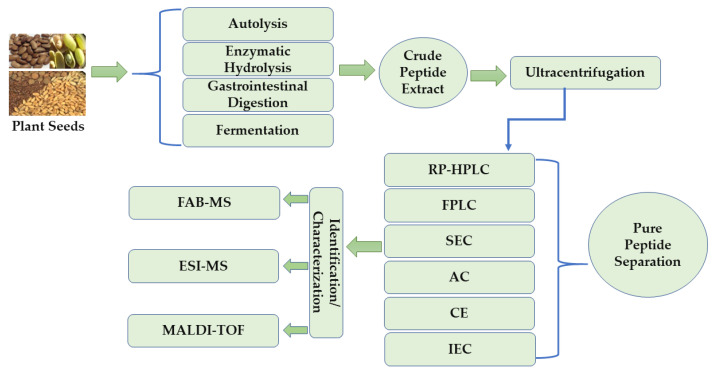
Schematic representation of the production, purification, and characterization of bioactive peptides from plant seeds. RP-HPLC—reversed-phase high-performance liquid chromatography, FPLC—fast protein liquid chromatography, SEC—size exclusion chromatography, AC—affinity chromatography, CE—capillary electrophoresis, IEC—ion-exchange chromatography, FAB-MS—fast atom bombardment mass spectrometry, ESI-MS—electrospray ionization mass spectrometry, MALDI-TOF—matrix-assisted laser desorption/ionization time-of-flight mass spectrometry.

BAPs derived from plant seeds have shown various biological activities ranging from antimicrobial to human physiological effects. These BAPs exist in a compounded mixture containing various biomolecules, which make their isolation and characterization a difficult task. The techniques primarily involved in BAP production usually lead to the generation of crude products that contain hydrolyzed peptides mixed with raw material residues [62]. Therefore, the crude hydrolysates require further purification processes to remove unwanted compounds and obtain fractions enriched with target peptides. To achieve the desired purification/separation purpose, the crude protein hydrolysate is commonly subjected to single or multiple chromatography, ultrafiltration, and ultracentrifugation methods [63]. In the first step of purification, crude hydrolysates are subjected to high-speed centrifugation, which may be followed by ultrafiltration with membranes of desired molecular weight cut-off [35,36,51]. Subsequently, the fractionated peptides are subjected to different modes of chromatographic methods such as ion exchange, size exclusion (SEC), and hydrophobic interaction chromatography. Commonly used instrumental methods that have been found to have strong potential in the separation/purification of peptides include capillary electrophoresis, fast protein liquid chromatography (FPLC), and reverse-phase high-performance liquid chromatography (RP-HPLC) [64]. González-García et al. [65] evaluated the isolation of BAPs from plum (*Prunus Domestica* L.) seed hydrolysate and were able to identify these peptides using RP-HPLC-MS/MS. Similarly, Hong et al. [66] purified a BAP from Chinese leek seeds using successive chromatographic techniques such as SEC and HPLC to obtain a BAP fraction (CLP-2). After fractionation, the purity of the bioactive peptides is routinely determined using mass spectrometry (MS) techniques. Electrospray ionization mass spectrometry (ESI-MS) is used for the analysis of polar compounds such as proteins. In contrast, matrix-assisted laser desorption ionization mass spectrometry (MALDI-MS) is highly effective in characterizing complex protein mixtures extracted from different sources, including edible plant seeds [35,62]. In addition, sequencing is performed using tandem MS (MS/MS) or database search to identify and determine the molecular weight of the peptides [66,67,68]. Sandoval et al. [68] conducted their study on chia (*Salvia hispanica* L.) seed protein. After fractionating chia seed protein via HPLC, fractions were subjected to tandem mass spectrometry (LC/ESI-MS/MS) for identification. Recently, the shotgun peptidomics method has shown promise for comprehensively identifying hundreds or thousands of peptides in crude protein hydrolysates without further downstream processing [69]. Figure 1 shows a schematic representation of the production, purification, and characterization of BAPs from edible plant seeds.

## 5. Bioactive Peptides from Edible Seeds and Their Roles in Human Health and Disease

Recent scientific evidence suggests that the role of proteins derived from food is not only to work as ordinary nutrients, but they also play crucial roles to modulate physiological functions. The discovery of biologically active compounds from dietary sources is becoming an exciting field of research with enormous potential. The growing consumer awareness regarding the impact of food on health is being reflected in the increasing demand for natural products, such as polyphenols, probiotics, minerals, and proteins. Edible seeds are an essential part of the human diet due to their high nutrient density. The seeds are rich sources of proteins, lipids, carbohydrates, minerals, and other micronutrients. The abundance of seed storage proteins makes them attractive for research as a rich source of BAPs with different health benefits. The AA sequences of bioactive peptides play a key role in their ability to modulate physiological functions of the body [70]. A growing tendency and research interest are to use the food protein-derived peptides as a tool for disease prevention and to maintain general well-being and health.

### 5.1. Antioxidant and Anti-Inflammatory Activities

It is a well-established fact that oxidative stress is a leading cause of many different diseases, including cancer, diabetes, obesity, and heart disease. The reason behind oxidative stress is imbalanced production and inadequate removal of reactive oxygen species (ROS) within cells. During normal cellular metabolism, ROS are generated to play some physiological roles such as signal transduction between cells [71]. However, certain types of molecules, pathological conditions, or environmental conditions, such as exposure to ionizing radiation or ultraviolet radiation, can increase ROS production within the human body. This increased level of ROS could lead to severe neurological disorders such as Alzheimer’s disease [72] or cause cancer through lethal damage to the DNA. Therefore, antioxidant compounds play an important role in preventing unwanted consequences of ROS or free radicals in the body. Efforts have been made in recent years to understand the role of proteins derived from various edible seeds as sources of peptides with the ability to reduce oxidative stress. An extensive number of antioxidant peptides have been identified from animals, plant, and insect sources, but the emphasis here will be on plant sources, especially edible seeds.

Antioxidant peptides are generally low molecular weight oligopeptides made up of 2–20 AA residues. It has been reported that peptides with more hydrophobic and aromatic AAs have strong antioxidant activity, though additional scientific evidence is needed for clarification. Antioxidative properties of the peptides were reported to be more related to their structure, composition, and other factors. Histidine, cysteine, lysine, methionine, tryptophan, and tyrosine are examples of AAs instrumental for strong antioxidant activity, while AAs having aromatic residues can donate electrons to electron-deficient radicals [21,22]. A recent study reported the sequence of BAPs that were present in mung bean meal protein hydrolysate (MMPH), and their antioxidative properties were confirmed. In the study, twelve peptides were identified with antioxidative capacity, i.e., QFAW, FLQL, EYW, ERF, QFAAD, MMGW, CSGD, LAF, CTN, LAN, CGN, and HC. The results confirmed that CSGD, CGN, HC, and CTN showed the most DPPH (2,2-diphenyl-1-picrylhydrazyl) radical scavenging activity with 0.30, 0.28, 0.29, and 0.30 mg/mL of EC_50_ values, respectively. EC_50_ is the half-maximal effective concentration, which means concentrations at which the substance/sample/drug is expected to produce 50% of the effect. Moreover, the findings also showed that CSGD, CGN, HC, and CTN had the most potent superoxide and hydroxyl radical scavenging activities [73]. The protein hydrolysate from defatted peanut kernel protein was also reported to show antioxidative properties. The results found that the pea protein hydrolysate obtained after treatment with esperase showed the highest antioxidative capacity with regard to linolenic acid peroxidation when compared to other proteases such as protease N, protease A, pepsin, and neutrase [74]. Alcalase and thermolysin-treated cherry seed protein hydrolysates/peptides showed the most potent ACE-inhibitory activity with antioxidant activity compared to the sample produced with Flavourzyme [75]. The results demonstrate the potential of food-derived peptides in mitigating oxidative reaction-induced damages in biological and food matrices. A recent study by Idowu et al. [76] assessed the sesame seed protein hydrolysate produced using pepsin and pancreatin enzymes for their effective antioxidant potential. The results confirmed that sesame seed protein hydrolysate had the highest hydroxyl radical scavenging and metal chelation activity compared to the unhydrolyzed protein. Recent in vitro and in vivo studies have shown that several BAPs derived from different sources have anti-inflammatory activity. However, the mechanisms by which these peptides work are still emerging. Tumor necrosis factor alpha (TNF-α) is an inflammatory cytokine liable for different signaling actions within the cells. When talking about interleukins (ILs), these are groups of proteins that mediate cell communications and arouse the immune response. Furthermore, IkB kinase is an enzyme complex that takes part in cellular reaction to inflammation. In addition, JNK (c-Jun *N*-terminal kinase) also works as an important signaling pathway to control various cellular processes. Another peptide, ɣ-glutamyl-cysteine, isolated from various food sources, including edible beans, inhibited the phosphorylation of IkB and JNK, unlike the mode of action by another peptide, VPY, which inhibited the secretion of IL-8 and TNF-α [77]. Another recent study found that the peptide fraction from globulin 7S derived from millet grains heated at 65 °C had potent inhibitory activities against cyclooxygenase-1 (COX-1, IC_50_ = 0.08 mg/mL) and cyclooxygenase-2 (COX-2, IC_50_ = 0.12 mg/mL) [78]. The latest study by Gao et al. [79] evaluated lupin peptide anti-inflammatory properties. This study produced lupin peptides from lupin proteins through gastroduodenal digestion and further identified them using ultraperformance liquid chromatography–tandem mass spectrometry. IQDKEGIPPDQQR, a lupin-derived peptide, was assessed by macrophage inflammatory cytokine production assay. The results showed that the lupin peptide inhibited monocyte chemoattractant protein-1, IL-1β, IL-6, and TNF-α production by 40.43, 44.70, 38.52, and 51.20%, respectively. Moreover, RNA-sequencing results also confirmed anti-inflammatory potentials of the lupin peptide. Previous research has reported the properties of amaranth-derived anti-inflammatory peptides. Peptides were obtained using simulated gastrointestinal digestion of germinated amaranth, and then collected after 90 min of incubation with pancreatin. Moreover, peptides were separated into <3 kDa, 3–10 kDa, and >10 kDa fractions. The results found that the 3–10 kDa and >10 kDa peptides showed a high anti-inflammatory response in RAW 264.7 macrophages [80]. Hemp seeds also had a wide variety of biologically active compounds. In a recent study, the anti-inflammatory potential of hemp seed protein hydrolysate (HPH) was reported using LPS-stimulated human primary monocytes. Lipopolysaccharides (LPS) are the major outer membrane component of Gram-negative bacteria, mainly lipids and polysaccharides with demonstrated toxic activity. The results of ELISA and RT-qPCR assays confirmed the anti-inflammatory properties of HPH. The results showed that HPH increased the levels of anti-inflammatory cytokines (IL-4 and IL-10) and reduced those of pro-inflammatory cytokines (IL-6, IL-1β, and TNF-α). Furthermore, the results confirmed that M1 polarization marker (CCR7 and iNOS) and M2 polarization marker (MRC1 and CD200R) gene expressions were downregulated and upregulated, respectively [81]. Millet seed peptides have also been reported for their beneficial bioactive functions. A recent study by Hu et al. [82] reported peptides’ anti-inflammatory and antioxidative potential derived from heated and germinated foxtail millet. In vitro GIT conditions were used to obtain bioactive peptides and the findings confirmed seven novel peptide sequences from the boiled germinated millet samples. The work found that seven peptides considerably (*p* < 0.05) reduced ROS production and enhanced superoxide dismutase activity and glutathione content in Caco-2 cells. Furthermore, the results confirmed that two peptides (QNWDFCEAWEPCF and EDDQMDPMAK) inhibited the production of TNF-α, nitric oxide, and IL-6 in RAW 264.7 cells. Table 1 shows examples of BAPs and protein hydrolysates from edible seeds and their reported antioxidant and anti-inflammatory activities.

### 5.2. Antihypertensive Activity

Hypertension is one of the biggest public health concerns worldwide. It is diagnosed when systolic and diastolic blood pressures rise above >140 and 90 mmHg, respectively [83]. Obesity, diabetes, and kidney diseases are well-established causes of hypertension. Disorders involving the renin–angiotensin system (RAS) are also causative factors in the development of hypertension. Within the RAS, ACE plays a pivotal role by converting inactive angiotensin I to the vasoactive angiotensin II. Therefore, excessive activities of ACE can lead to strong vasoconstrictions with weak vasorelaxation, and hence high blood pressure develops. The inhibition of ACE activity by drugs is a proven pharmacological approach for lowering blood pressure. However, the adverse side effects associated with current antihypertensive ACE-inhibitory drugs could lead to a compromised state of health of the patient with a significant negative impact on the quality of life or life expectancy and healthcare costs. Apart from ACE regulation, newer approaches propose that food-derived peptides exhibit antihypertensive potential via ACE2 (an ACE homologue) upregulation, which balances the harmful outcome of raised ACE and leads to decreases in vascular oxidation and inflammation along with the enhancement of endothelial function [84]. Therefore, there is interest in using natural ACE inhibitors such as plant-based bioactive peptides to replace or complement drugs as antihypertensive agents [85]. In a separate study, Malomo et al. [86] showed that the protein hydrolysate from hemp seed possesses antihypertensive activities. In their study, they used 2% papain, 2% alcalase, 1% alcalase, 2% pepsin, 4% pepsin, and 2% pepsin + pancreatin to digest the hemp seed proteins. The resultant hemp seed protein hydrolysates (HPHs) were administered orally to spontaneously hypertensive rats (dosage of 200 mg/kg body weight) followed by SBP measurement for 24 h. The results showed that the 1% alcalase HPH treatment was highly effective in lowering the SBP efficiently (−32.5 ± 0.7 mmHg after 4 h). In contrast, the pepsin hydrolysate had a long-lasting effect (−23.0 ± 1.4 mmHg after 24 h). Ma et al. [87] conducted a study on the novel ACE-inhibitory peptides from *Ginko biloba* seeds. They used LC-MS/MS and identified three novel ACE-inhibitory peptides: TNLDWY (IC_50_ = 1.932 mM), RADFY (IC_50_ = 1.35 mM), and RVFDGAV (IC_50_ = 1.006 mM).

**Figure 2:**
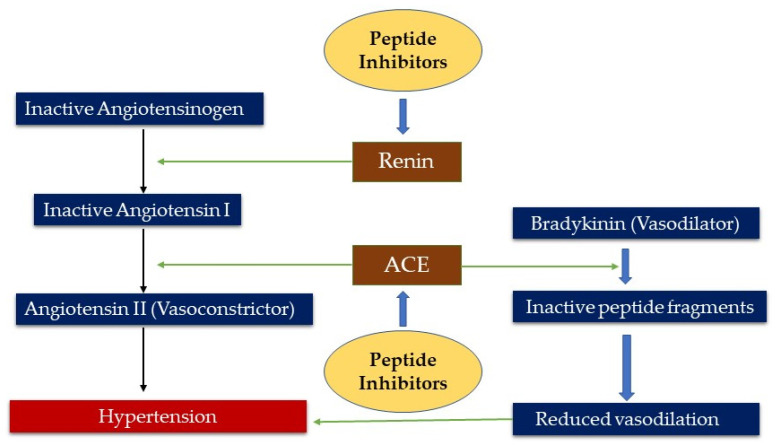
This schematic diagram shows the mechanism by which blood pressure is controlled and the probable targets of antihypertensive peptides.

Previous works have shown that partial enzymatic hydrolysis of plant proteins could produce a broad range of ACE-inhibitory peptides. Huang et al. [88] showed that ultrasound pre-treated wheat germ protein produced an enzymatic protein hydrolysate with 32% higher ACE-inhibitory activity when compared to the non-pretreated protein. In another study, Chao et al. [89] showed that high pressure (600 Mpa) pretreatment of pea proteins led to a significant increase in ACE-inhibitory activity after hydrolysis with 1% alcalase. In a similar study, alcalase and thermolysin hydrolysates of pitted plum seed proteins showed higher ACE-inhibitory activity compared to Flavourzyme and protease P-treated samples [65]. In several studies, in vitro inhibition of ACE activity by edible seed-derived peptides has resulted in blood pressure lowering effects in animal models and humans [67,86,87]. For instance, oral administration of a black soybean peptide mixture to prehypertensive patients for eight weeks significantly reduced SBP. In addition to ACE inhibition, this effect was reported to be mainly due to the high arginine content of the sample [90]. The reason behind this is that arginine is a precursor of nitric oxide, which possesses vasodilatory activity and also acts as an ACE inhibitor. Renin is another target to control hypertension because this enzyme generates angiotensin I from angiotensinogen. Three dipeptides (IR, KF, and EF) from a digest of pea protein were reported to have renin-inhibitory activity. The renin-inhibitory activity of peptides with positively charged AAs was more effective than those carrying negatively charged AAs [91]. In a previous study, wheat bran protein hydrolysate was produced with alcalase and fractionated into different peptide sizes using membrane ultrafiltration. Furthermore, these hydrolysates were evaluated for inhibition of ACE and renin activities. The results showed that renin and ACE inhibition were considerably higher for the <1 kDa fraction, i.e., 75.19% ± 1.75%, and 84.25% ± 2.45%, respectively. Moreover, the results confirmed that the <1 kDa membrane fraction (100 mg/kg weight), when orally administered to spontaneously hypertensive rats, decreased (−35 mmHg) the SBP [92]. A recent study by Aondona et al. [93] assessed the in vitro antihypertensive potential of sesame seed protein hydrolysate and its fractionated peptides. The sesame seed protein hydrolysate was formed by using pepsin and pancreatin and then filtered through membrane ultrafiltration in order to obtain different peptide size fractions. The results found that the <1 kDa peptide fraction was the most potent ACE inhibitor (81%), while peptides having 5–10 kDa and 3–5 kDa sizes were the most potent (75–85%) renin inhibitors. The study findings suggest that sesame seed protein hydrolysate products could serve as potential antihypertensive agents. Another work by Udenigwe et al. [94] confirmed the potential activities of renin-inhibiting dipeptides using quantitative structure–activity relationship modeling. The modeling results suggested that low molecular size AAs with hydrophobic side chains were favored at the *n*-terminus. In contrast, AAs with bulky side chains were favored at the C-terminus for renin inhibition potency. Another study reported that peptides TF, LY, and RALP with renin-inhibitory activity were isolated from an alcalase digest of rapeseed protein; LY and RALP with predominantly hydrophobic groups had better activity than TF [95]. Lastly, hemp seed protein-derived peptides WYT and SVYT showed 76% and 86% in vitro renin-inhibitory activity, respectively, at 0.5 mg/mL peptide concentration [48]. Though these peptides have shown promising in vitro results, sometimes they are ineffective under in vivo conditions. This may be due to the activity of gastrointestinal enzymes, which degrade these peptides to limit absorption and bioavailability [11,96]. As protein hydrolysates contain a mixture of different peptides, the identification of specific peptides remains a challenge. Apart from the limitations of GIT inactivation during in vivo testing and the high cost of purification, peptides have enormous potential as antihypertensive agents. Figure 2 shows the mechanism by which blood pressure is controlled and the probable targets of antihypertensive peptides derived from edible seeds. Table 2 shows examples of BAPs/hydrolysate from edible seeds and their reported physiological functions (antihypertensive activity).

### 5.3. Hypoglycemic Activity

Diabetes is a very old disease of human civilization. Different effective therapeutic approaches have been successfully used during the last few years, but a lot of side effects of drug treatments may compromise patients’ health. BAPs and protein hydrolysates from different edible seeds possess antidiabetic activity and have the potential to be used as an alternative therapy. Peptides exert antidiabetic effects through one or more mechanisms, such as the inhibition of dipeptidyl peptidase IV (DPP-IV), leading to a reduced blood glucose level and an increased physiological level of insulin. Various edible seeds are used as protein sources to isolate antidiabetic hydrolysates and peptides through enzymatic digestion [97]. Apart from DPP-IV inhibition, the administration of natural constituents/ingredients that have the capacity to inhibit α-glucosidase and α-amylase activities is also another significant approach to control diabetes. This is because α-glucosidase and α-amylase are the main digestive enzymes that take part in the metabolism of dietary carbohydrates. These enzymes break down digestible carbohydrates to release free glucose, which is then absorbed into the body. Evidence has been established that food-derived peptides contain attributes that regulate the activities of α-glucosidase and α-amylase [98]. A recent study by Olagunju et al. [99] reported the in vitro anti-diabetic potential of pigeon pea peptides. In the study, pigeon pea protein was hydrolyzed with thermoase and the hydrolysate was separated into different peptide fractions (<1, 1–3, 3–5, 5–10, and >10 kDa) using ultrafiltration. In vitro results found that all the peptide fractions inhibited α-amylase and α-glucosidase activities, with the 3–5 and >10 kDa peptides being the most potent.

Hatanaka et al. [100] reported that rice bran protein hydrolysate had the capability of inhibiting DPP-IV activity. For this study, the authors used two enzymes (Bioprase SO and Umamizyme G) and showed that the rice bran peptides generated using Umamizyme G had ten times more inhibitory activity than those from Bioprase SP. The IC_50_ value of the rice bran peptide was 2.3 ± 0.1 mg/mL. In a different study to evaluate the potential of antidiabetic peptides from two rice products, rice bran and sake lees were subjected to enzymatic digestion with a commercially available protease (Denazyme AP) [101]. The results demonstrated that the protein hydrolysates generated from the rice bran showed higher DPP-IV inhibition activity when compared to the hydrolysate from sake lees. Previous evidence also supports the anti-diabetic properties of soybean and lupin protein hydrolysates. Six peptides (GQEQSHQDEGVIVR, LILPKHSDAD, LTFPGSAED, YVVNPDNNEN, YVVNPDNDEN, and IAVPTGVA, respectively) were assessed for their potential to inhibit DPP-IV activity. The results found that IAVPTGVA (soybean) and LTFPGSAED (lupin) are the most effective inhibitors with IC_50_ values of 106 and 228 μM, respectively [102]. Furthermore, another study confirmed the α-amylase inhibition characteristics of five peptides obtained from pinto bean (PB), using pancreatic cell line AR42J. The results found that PB peptide 9 (LSSLEMGSLGALFVCM) showed the highest α-amylase inhibitory activity [103]. In a recent study by González-Montoya et al. [104], the anti-diabetic potential of germinated soyabean peptides was confirmed. In the study, a protein hydrolysate was produced using pancreatin and pepsin and further separated into peptide fractions of <5, 5–10, and >10 kDa sizes using ultrafiltration. Subsequently, peptides were assessed for in vitro inhibiting sucrase, maltase, α-amylase, and DPP-IV activities. The results reported that the <5 and 5–10 kDa peptides significantly inhibited α-glucosidase and α-amylase activities, whereas >10 and 5–10 kDa peptides were inhibitors of DPP-IV (IC_50_ = 1.18 and 0.91 mg/mL, respectively).

Amaranth grain contains storage proteins such as globulins, glutelins, and albumins, which were subjected to enzymatic hydrolysis using alcalase to produce hydrolysates. The results confirmed that albumin 1, globulin, and glutelin hydrolysates competitively inhibited DPP-IV activity in vitro with an inhibitory constant (Ki) of 0.11–5.61 mg/mL. Furthermore, the evidence established that the glutelin hydrolysates possessed the highest enzyme inhibitory activity [105]. In another study, the protein hydrolysates were separated by SEC and collected fractions orally administered to diabetic mice. The results showed that a fraction of glutelin hydrolysate had the highest DPP-IV inhibitory activity [106]. Quinoa is a pseudo-cereal, which contains a higher protein content than most cereals; therefore, quinoa seeds have been used as a rich source of BAPs. Peptides released through a simulated gastrointestinal digestion of quinoa seed proteins showed antidiabetic activities [107]. Notably, peptides from the enzymatic digestion of quinoa seed proteins with a molecular weight lower than 5 kDa showed high α-glucosidase inhibitory activity with an IC_50_ value of 1.45 ± 0.12 mg protein/mL. In another study, oat-derived peptides were evaluated for hypoglycemic potential using a streptozotocin-induced diabetic mice model. Different doses of oat peptides (0.25, 0.5, and 1.0 g kg/bw) were orally administered to mice for four weeks. The results found that mice that received the highest oat peptide dose had a considerably higher insulin activity index, serum fasting insulin, and food efficiency but significantly lowered fasting blood glucose and total food intake compared to the other groups that received lower doses [108].

Mojica et al. [109] studied peptides generated by enzymatic digestion from Mexican black beans and Brazilian carioca. Four peptides had strong α-glucosidase and DPP-IV inhibitory activity, with CPGNK, KTYGL, GGGLHK, and KKSSG inhibiting DPP-IV activity at IC_50_ values of 0.87, 0.03, 0.61, and 0.64 mg dry weight/mL, respectively. Mojica et al. [110] conducted a study and evaluated the hypoglycemic potential of a hydrolyzed protein from black beans and peptides purified using in vitro and silico analysis. The results showed that the black bean fractions significantly reduced blood sugar levels by blocking glucose transporters in Caco-2 cells. Rocha et al. [111] also demonstrated that germination and alcalase hydrolysis influenced the antidiabetic potential of peptides isolated from black bean proteins. The study showed that neither the insulin secretion nor the DPP-IV inhibition was improved, but germination after 24 h increased the α-amylase inhibitory activity of the sample. After simulated GIT digestion of the black bean proteins, the peptides generated also had the potential to increase insulin secretion. This suggests that endogenous proteases could release BAPs with health promoting effects. A previous study by Oseguera-Toledo et al. [112] confirmed the anti-diabetic capacity of isolated peptides from two common beans (pinto Durango and black 8025). Aglycin is a natural 37-amino acid residue bioactive peptide (ASCNGVCSPFEMPPCGSSACRCIPVGLVVGYCRHPSG), which was isolated from soybean. Aglycin has a stable structure (during enzymatic action) and three disulfide bonds involving six cysteine AAs. Furthermore, it has been reported that aglycin was found to be effective in reducing blood glucose in diabetic mice when orally administered at 50 mg/kg body weight/d for four weeks [113]. A recent study by Boachie et al. [114] assessed the pigeon pea protein inhibition of DPP-IV activity using in vitro and in silico parameters. In silico results confirmed that 46% of AAs in 40 pigeon pea proteins possessed DPP-IV inhibition attributes. Furthermore, in vitro findings exhibited that thermolysin liberated the most active DPP-IV inhibitors. The overall finding of the study established that pigeon pea proteins contain DPP-IV inhibitory peptide sequences, which could be used as promising functional food ingredients for the management of type 2 diabetes. The above evidence suggests that edible seed peptides possess the potential attributes to regulate several activities such as dipeptidyl peptidase DPP-IV, α-glucosidase, and α-amylase, which could be used to control diabetes and related conditions. Table 3 shows examples of edible seed-derived BAPs and protein hydrolysates with potential hypoglycemic activity.

### 5.4. Anti-Cancer Activity

One of the main problems with the available cancer treatments is the severe negative side effects of drugs used as chemotherapeutic agents. Bioactive peptides have potential cytotoxic activity in different cancer cell lines and could be used as anti-cancer agents with minimal or no side effects. Peptides could act as cytotoxic components through different mechanisms. Additionally, BAPs could work as new carriers of cytotoxic agents to target cancer cells specifically without killing normal cells [115,116]. Peptides might inhibit specific molecular signaling pathways of cancer cells related to the process of oncogenesis, cancer stem cell self-renewal, and differentiation pathways. Different types of dysregulated signaling pathways could be targeted by specific peptides. Previously reported evidence has confirmed the inhibiting effects of mung bean, adzuki bean, black soybean, and soybean meal proteins against cancer cells (hepatocellular carcinoma cells SMMC-7721 and ovarian cancer cell line SKOV3) in the concentration range of 200–600 g/mL [117]. In addition, another study reported the anti-cancer potential of quinoa peptides, i.e., IFQEYI, DVYSPEAG, RELGEWGI (F-3), DKDYPK (F-2), and LWREGM (F-1), using human colorectal cancer cell lines [118]. Taniya et al. [119] also reported the anti-cancer property of an amaranth seed protein hydrolysate. The hydrolysate was formed by simulated GIT digestion and further evaluated for anti-cancer effect using an in vitro breast cancer cell model. The results confirmed that the digested amaranth sample inhibited the growth of cells with 50% growth inhibition concentration of 48.3 ± 0.2 μg/mL.

Lunasin is a soybean-derived peptide that possesses antineoplastic effect in different cancer cell lines through various mechanisms [120]. Studies have reported the anticarcinogenic activity of lunasin in vivo, especially the potential protective effect on breast cancer in mice. Tumor occurrence was 67% and 50%, respectively, for the control and experimental diet-fed mice after AIN-93G supplementation with 0.23% lunasin (lunasin-enriched soy protein concentrate) [121]. Another research study by Devapatla et al. [122] assessed the chemoprotective and therapeutic potential of lunasin as an anti-cancer agent. In this study, lunasin potential was evaluated using in vitro colony formation and cell proliferation assays against murine Lewis lung carcinoma (LLC) and B16-F0 melanoma cells. In addition, in vivo tests used C57BL/6 mice (lunasin-treated and untreated), which were subcutaneously implanted with B16-F0 or LLC cells. The results showed that lunasin inhibited the growth of murine B16-F0 melanoma cells and LLC cells in vitro and in the C57BL/6 mice model.

Another group of plant-derived proteins with anti-cancer properties is lectins. Lectins recognize and bind with specific carbohydrate moieties on cancer cells. A tepary bean lectin was found to be effective against different human cancer cell lines, such as cervical and colon cancer cell lines [123]. A previous study reported the anti-tumor potential of an olive seed-derived peptide (LLPSY). The results confirmed that LLPSY had anti-proliferative potential in a dose-dependent manner when tested on MDA-MB-468 and PC-3 cancer cells [124]. Another piece of evidence suggested the anti-cancer property of chickpea peptide using MDA-MB-231 and MCF-7 cell lines with RQSHFANAQP inhibiting the growth of breast cancer cell lines via p53 protein [125]. Previous evidence confirmed that a rapeseed peptide extract, which was prepared using mixed solid-state fermentation, had anti-proliferative properties when tested against human HeLa cervical, MCF-7 breast, and HepG2 liver cancer cell lines [126]. Table 4 shows examples of BAPs and protein hydrolysates from edible seeds with demonstrated anti-cancer activity.

### 5.5. Mineral-Binding Peptides

Minerals such as iron, zinc, and calcium are essential inorganic micronutrients that play vital biological roles in maintaining human health [127,128]. Thus, mineral deficiencies are a public health concern worldwide. The nutritional value of raw foods is lower due to reduced mineral bioavailability, which could cause several metabolic health issues. Hence, the nutritional status of a population could be promoted through increasing the nutrient bioavailability of foods [129]. For instance, iron is an integral part of erythrocyte hemoglobin, muscle myoglobin, liver ferritin, and serves as a cofactor for several cellular enzymes. To prevent or alleviate health issues associated with mineral deficiencies, ion-binding peptides from plant seeds can be used as promising carriers of mineral supplements. Some organic compounds, such as amino acid chelators, that form complexes with metal ions have reduced mineral interactions with the food matrix [130,131]. For example, ferrous bis-glycinate was found to result in four times higher iron absorption than ferrous sulphate because the bis-glycinate effectively protected iron from the inhibitory effect of maize phytate in vivo [132]. Due to the excessive cost of AAs, peptides derived from edible seed proteins commonly produced in vivo or in vitro by enzymatic proteolysis are promising ligands for complexation with divalent metals towards improving mineral bioavailability and for alleviating micronutrient deficiency [130,133,134]. To date, a variety of metal-chelating peptides have been generated and identified from different edible seed proteins [135,136]. For instance, Wang et al. [137] reported that a pentapeptide FVDVT from wheat germ protein hydrolysate (WGPH) possessed significantly (86%) higher calcium-binding property when compared to crude hydrolysates. The authors proposed that the amido group nitrogen atoms and carboxy group oxygen atoms were the major metal-binding sites. Mineral-chelating peptides possess a structurally diverse backbone as they contain both the terminal carboxyl and amino groups and the side chains of AA residues. Furthermore, the cysteine, glutamic acid, serine, and aspartic acid residues that contain S, O, and *N* atoms contribute to the construction of peptide–mineral complexes [130,134,136]. For instance, the serine hydroxyl group has a robust binding capacity with zinc [138]. Moreover, another piece of evidence established that the WGPH had nearly 69.62% metal-chelating ability. The zinc-chelating HNAPNPGLPYAA and NAPLPPPLKH were identified as the two major peptides in the WGPH using MALDI TOF/TOF. Furthermore, HNAPNPGLPYAA had a higher (~92%) zinc-chelating capacity and higher bioavailability of zinc in Caco-2 cells than ZnSO_4_-treated cells [139]. In addition, wheat germ protein was found to be a potential source for calcium-binding peptides. For example, peptides produced from hydrolyzed wheat germ protein at 21.5% degree of hydrolysis using alcalase had improved calcium binding capacity. It was suggested that the calcium-binding regions of peptides are mainly comprised of glycine, aspartic acid, arginine, and glutamic acid [140]. Indeed, in addition to the carboxyl- and amino-terminal groups, AA side-chain groups such as guanidine (arginine), ε-amino (lysine), sulfhydryl (cysteine), hydroxyl (serine), and carboxyl (glutamic acid and aspartic acid) are responsible for metal binding [134]. Lv et al. [141] also reported the isolation of iron-chelating peptides VEDELVAVV and LAGNPDDEFRPQ from defatted walnut flake. The iron and calcium-binding capacities of mung bean-derived peptides were assessed by Budseekoad et al. [142]. Enzymatically hydrolyzed mung bean hydrolysates were separated into different fractions using ultrafiltration and SEC. The results found that fraction 2 had the most potential iron and calcium-binding abilities. RP-HPLC separation coupled with mass spectrometry identified several peptides, among which HADAD, AIVIL, and LLLGI had the best calcium-binding capability, whereas PAIDL had the most effective iron-binding capacity. Another study also confirmed the potential of mung bean protein-derived peptides for iron-binding capacity [143]. Using an enzymatic membrane reactor, the mung protein hydrolysate was prepared, and further peptide separation was carried out by anion-exchange chromatography and reverse-phase HPLC. The results showed that the PAIDL peptide had significantly the highest iron-binding ability. Table 5 shows examples of mineral-binding BAPs and protein hydrolysates from edible seeds.

## 6. Concluding Remarks and Future Perspectives

Edible seed-derived BAPs have immense potential to interact with cells, tissues, enzymes, and other target molecules to exhibit several therapeutic attributes useful in regulating and treating lifestyle-related health disorders, including cancer, diabetes, hypertension, and inflammation. This review provided an overview of BAPs derived from edible seeds with multifunctional characteristics related to human health promotion. Research on BAPs is attracting the interest of scientists due to the discovery of several molecular targets to ameliorate human lifestyle. However, other concerns, including scale-up challenges, potential peptide reactivity with the food matrix, biostability (structural breakdown during passage through the GIT), potential bitter taste, and the safety of BAPs, need to be properly addressed prior to their incorporation into human food products. Therefore, it is necessary to obtain strong evidence using human studies on different potential concerns, as mentioned above, to enhance the use of BAPs for the development of nutraceuticals and functional foods.

## Figures and Tables

**Table 1:** Antioxidant and anti-inflammatory activities of bioactive peptides and protein hydrolysates from edible seeds.

Seeds	Bioactive Peptides/Protein Hydrolysates	Activities	References
Mung bean	CSGD, CGN, HC, and CTN	Radical scavenging activity (antioxidative properties)	[73]
Peanut kernels	Protein hydrolysate	Antioxidative capacity with regard to linolenic acid peroxidation	[74]
Cherry seed	Protein hydrolysates/peptides	Angiotensin-converting enzyme (ACE)-inhibitory activity	[75]
Sesame seed	Protein hydrolysate	Ferric reducing power (antioxidant) and metal chelation activity	[76]
Lupin	IQDKEGIPPDQQR (IQD) lupin-derived peptide	Inhibited monocyte chemoattractant protein-1, interleukin IL-1β, (IL)-6, and tumor necrosis factor-α production	[79]
Amaranth	<3 kDa, 3–10 kD, and >10 kDa peptide fractions	Anti-inflammatory response in RAW 264.7 macrophages	[80]
Hemp seed	Protein hydrolysate	Increased *IL-4* and *IL-10* levels and reduced *IL-6, IL-1β,* and *TNF-α* levels	[81]
Foxtail millet	QNWDFCEAWEPCF and EDDQMDPMAK	Inhibited tumor necrosis factor-α, nitric oxide, and IL-6 production in RAW 264.7 cells	[82]

**Table 2:** Antihypertensive bioactive peptides and protein hydrolysates from edible seeds.

Seeds	Bioactive Peptides/Protein Hydrolysates	Activities	References
Hemp seed	Protein hydrolysates	Lowered the SBP in hypertensive rats	[86]
*Ginko biloba*	TNLDWY, RADFY, and RVFDGAV	ACE-inhibitory activities	[87]
Wheat bran	Protein hydrolysates	Renin and ACE inhibition activity	[92]
Sesame seed	Different peptide fractions	Anti-hypertensive potentials	[93]
Rapeseed	TF, LY, and RALP	Renin-inhibitory activity	[95]

**Table 3:** Hypoglycemic activity of bioactive peptides and protein hydrolysates from edible seeds.

Seeds	Bioactive Peptides/Protein Hydrolysates	Activities	References
Pigeon pea	Peptide fractions (<1, 1–3, 3–5, 5–10, and >10 kDa)	Inhibited α-amylase and α-glucosidase	[99]
Defatted rice bran	UG peptides	DPP-IV inhibition activity	[100]
Soybean and lupin	IAVPTGVA (soybean) and LTFPGSAED (lupin)	Inhibited DPP-IV activity	[102]
Amaranthus grain	Albumin hydrolysate fraction after 48 h (AHF48)	DPP-IV inhibition activity	[105]
Quinoa	Bioactive peptides IQAEGGLT, DKDYPK, GEHGSDGNV	Anti-diabetic activity, free radical scavenging activity	[107]
Oat	Oat peptides (0.25, 0.5, and 1.0 g kg/bw)	Lowered fasting blood glucose	[108]
Black beans	Bioactive peptides (AKSPLFATNPLF, FEELN, LSVSVL)	Reduced glucose uptake, blockage of glucose transport, and DPP-IV inhibition activity	[110]
Common beans	Bioactive peptides (RGPLVNPDPKPFL)	Anti-diabetic activity (DPP-IV inhibition)	[111]

**Table 4:** Edible seed-derived bioactive peptides and protein hydrolysates with anti-cancer properties.

Seeds	Bioactive Peptides/Protein Hydrolysates	Activities	References
Quinoa	IFQEYI, DVYSPEAG, RELGEWGI (F-3), DKDYPK (F-2), and LWREGM (F-1)	Anti-cancer potential	[118]
Amaranth	Protein hydrolysate	Anti-cancerous effect using breast cancer cell model	[119]
Olive	LLPSY peptide	Anti-proliferative potential when tested on MDA-MB-468 and PC-3 cancer cells	[124]
Chickpea	RQSHFANAQP	Anti-cancerous property using MDA-MB-231 and MCF-7 cell lines	[125]

**Table 5:** Edible seed-derived mineral-binding bioactive peptides and protein hydrolysates.

Seeds	Bioactive Peptides	Activities	References
Wheat germ	Pentapeptide FVDVT	Calcium-binding property	[137]
Walnut flake	VEDELVAVV and LAGNPDDEFRPQ	Iron-chelating peptides	[141]
Mung bean	HADAD, AIVIL, and LLLGI	Calcium-binding capability	[142]
Mung bean	PAIDL	Iron-binding capacity	[143]
